# Prevalence of sleep disorders in Parkinson’s disease patients in two neurology referral hospitals in Ethiopia

**DOI:** 10.1186/s12883-019-1431-2

**Published:** 2019-08-22

**Authors:** Dereje Melka, Abenet Tafesse, James H. Bower, Demeke Assefa

**Affiliations:** 1 Department of Neurology, Addis Ababa University School of Medicine, P.O.Box 1176, Addis Ababa, Ethiopia; 2 Department of Neurology, Mayo Clinic School of Medicine, 200 First St SW, Rochester, MN USA; 3 Department of Health Service Management and Reproductive Health, School of Public Health College of Health Sciences, Addis Ababa University, Addis Ababa, Ethiopia

**Keywords:** Parkinson’s disease, Sleep disorders, Africa, Poverty

## Abstract

**Background:**

Non motor symptoms (NMS) of Parkinson’s disease (PD) are common and can be more disabling than motor symptoms. Sleep disorders can be seen in up to 98% of patients with Parkinson disease. Poor sleep quality has been associated with poverty and race, and yet there has been no prior report on sleep disorders in those with PD living in sub Saharan Africa. We wished to document the prevalence of sleep disorders in PD patients in Ethiopia.

**Methods:**

We conducted a cross-sectional point prevalence study from July 1 to October 30, 2015 of all patients attending the neurology outpatient department in Tikur Anbessa and Zewuditu Memorial Hospitals, Addis Ababa, Ethiopia. Demographic data, clinical history and physical examination findings were collected from participants using a structured questionnaire. We used the Parkinson’s disease sleep scale version two (PDSS-2) and Epworth Sleepiness Scale (ESS) to assess the sleep symptoms.

**Results:**

Of the 155 patients surveyed, all patients reported some sleep problem. Over 43.9% of patients had a PDSS score > 18. The median score of ESS was 9 (IQR = 5–12), with 77/155 (49.7%) of the patients having possible or definite excessive daytime somnolence. A high EDSS score significantly associated with a Hoehn & Yahr score > 4 (*p* = 0.02).

**Conclusions:**

In Ethiopian PD patients, the prevalence of those with severe sleep disorders is the highest reported to date. The prevalence of possible/definite EDS is amongst the highest in the world. Further investigation into whether poverty or race explains this finding is needed.

## Background

The NMS of PD are frequent and can be very disabling [1, 2].

Sleep disorders associated with PD are one of the most common NMS and have been reported in 38 to 98% of PD patients [3]. They were first mentioned by James Parkinson himself in his famous monograph about the disease. Sleep disorders can occur before the diagnosis of PD, but become more severe and frequent as the disease stage progresses [2].

Patients with PD are at a greater risk for developing sleep disturbances than the general population. Sleep disturbances are a common but often under recognized feature of PD in clinical practice because of the absence of systematic or specific questioning by health care professionals [4].

There have been limited recent studies published on PD in sub Saharan Africa [5–12] and far fewer from Ethiopia [13, 14]. Poor sleep quality has been found to be strongly associated with poverty and race [15] and yet there have been no published data on sleep disorders in people with PD living in sub Saharan Africa. We wanted to document the prevalence of sleep disorders and their determinant factors in people living with PD in Ethiopia.

## Methods

We conducted a cross-sectional point prevalence study from July 1 to October 30, 2015 of all patients attending the neurology outpatient department**s** in Tikur Anbessa and Zewuditu Memorial Hospitals in Addis Ababa University. These serve as the hospitals for the only neurology training center in Ethiopia. Inclusion criteria were patients ≥18 years old diagnosed with PD using the UK Parkinson’s Disease Society Brain Bank Clinical Diagnostic Criteria seen at the two referral hospitals during the study period who gave informed verbal consent for study participation. Exclusion criteria were secondary Parkinsonism or refusal of informed verbal consent. Demographic data, clinical history and physical examination findings were collected from participants using structured questionnaires in Amharic and English. We also used two data collection instruments: the Parkinson Disease Sleep Scale version- 2 (PDSS-2) [16], and the Epworth Sleepiness Scale (ESS) [17, 18].

The PDSS-2 is a scale addressing 15 commonly reported symptoms associated with sleep disturbance. This scale has been shown to correlate with the Parkinson’s disease Quality of Life Questionnaire (PDQ-39), the Unified Parkinson’s Disease Rating Scale motor scores (UPDRS-III), and the Clinical Global Impressions Severity Score (CGI Item 1) [19–21].

The ESS is used as a subjective measure of a patient’s daytime sleepiness. This scale has a list of eight situations in which patients rate their likelihood of becoming sleepy on a scale of 0–3. Total score ranges from 0 to 24. A score of 10–15 suggests possible excessive daytime somnolence, and a score of 16–24 suggests definite excessive daytime somnolence [17, 18].

The PDSS-2 and ESS were translated from English into Amharic and pilot-tested on 10 subjects. All subjects understood every question without difficulty. No re-translation was required. These subjects were not included in the study results.

Analysis was performed using SPSS/PC version 20.0 software packages for statistical analysis (SPSS). Descriptive summaries were employed to describe socio-demographic and clinical characteristics. Appropriate measures of central tendency, frequency distribution, cross tabulation, Fisher’s Exact test and binary logistic regression analysis were conducted. Odds ratios and 95% confidence intervals were calculated. A *p* value less than 0.05 was considered a statistically significant association between assessed variables.

Protocol approvals were obtained from the ethical review Committee of the Department of Neurology and the Institutional Review Board and Research and Publication Committee of the College of Health Sciences of Addis Ababa University. Informed patient consent was obtained verbally before study enrollment. Patient data was deidentified during subsequent analysis and dissemination.

## Results

Out of 158 patients who presented during the study period, two refused consent and one did not fulfill the diagnostic criteria. A total of 155 subjects were included in this study: 127 (81.9%) male: 28 (18.1%) female. Table 1 shows the demographics of our subjects. The mean duration of symptoms, duration since PD diagnosis and duration of PD treatment were 6.37, 4.90, 4.68 yrs. respectively. All patients were taking levodopa and 23.9% were taking trihexyphenidyl. No patient was taking other anti-parkinsonian agents (e.g. dopamine agonist, amantadine).

**Table 1:** Socio-demographic Factors

Variables	Numbers (%)
Gender	
Female	28 (18.1)
Male	127 (81.9)
Age	
< 60 years	89 (57.4)
≥ 60 years	66 (42.6)
Marital status	
Never married	6 (3.9)
Married	121 (78.1)
Widowed	19 (12.3)
Divorced/separated	9 (5.8)
Duration of PD symptoms in years	
< 5 years	85 (54.8)
≥ 5 years	70 (45.2)
Employment status	
Employed	44 (28.4)
Unemployed	111 (71.6)
Educational status	
No formal education	48 (31)
Primary education	45 (29)
Secondary education	36 (23.2)
More than secondary education	26 (16.7)
Hoehn and Yahr stage	
Stage 1	37 (23.9)
Stage 2	46 (29.7)
Stage 3	44 (28.4)
Stage 4	23 (14.8)
Stage 5	5 (3.2)
Previous history of sleep disorder	
Yes	37 (23.9)
No	118 (76.1)

Table 2 shows the results of the PDSS-2 scores. No patient had a score of zero (range 4–39). The median score was 17 (IQR 11–24). Overall, 66/155 (42.6%) reported not having slept well > 2 days per week. 68/155 (43.9%) scored > 18. The most frequent sleep problems (defined as > 2 nights per week) were due to nocturia (73.5%), followed by difficulty with mobility in bed (37.4%), distressing dreams (36.1%) and sleep maintenance insomnia (34.8%).

**Table 2:** Results of Parkinson Disease Sleep Scale Version 2

Questions	Very often (6–7 days/ week) No (%)	Often (4–5 days/ week) No (%)	Sometimes(2–3 days/ week) No (%)	Occasionally(1 day/ week) No (%)	Never No (%)
1. Overall did you sleep well during the last week?	89 (56.8)	19 (12.6)	19 (12.6)	23 (14.8)	5 (3.2)
2. Did you have difficulty falling asleep each night?	6 (3.9)	16 (10.6)	25 (16.1)	36 (23.5)	72 (45.8)
3. Did you have difficulty staying asleep?	11 (7.1)	19 (12.8)	24 (15.8)	32 (20.6)	69 (43.9)
4. Did you have restlessness of legs or arms at night or in the evening causing disruption of sleep?	4 (2.6)	23 (14.8)	14 (9.4)	34 (21.9)	80 (51.3)
5. Was your sleep disturbed due to an urge to move your arms or legs?	5 (3.4)	17 (11.4)	20 (12.9)	35 (22.6)	78 (49.7)
6. Did you suffer from distressing dreams at night?	12 (7.7)	16 (10.6)	28 (18.4)	30 (19.4)	69 (43.9)
7. Do you suffer from distressing hallucinations at night (seeing or hearing things that you are told do not exist)?	4 (2.6)	10 (6.7)	14 (9.4)	27 (17.4)	100 (63.9)
8. Do you get up at night to pass urine?	58 (37.6)	33 (21.3)	23 (14.8)	26 (16.8)	15 (9.4)
9. Did you feel uncomfortable at night because you were unable to turn around in bed or move due to immobility?	11 (7.4)	23 (14.8)	24 (15.5)	45 (29.3)	52 (32.9)
10. Did you feel pain in your arms or legs which wake you from sleep at night?	2 (2.6)	14 (9.0)	25 (14.2)	36 (24.5)	76 (49.7)
11. Did you have painful muscle cramps in your arms or legs which wake you from sleep at night?	2 (1.6)	14 (9.0)	19 (12.3)	48 (31.3)	72 (45.8)
12. Did you wake early in the morning with painful posturing of arms or legs?	4 (2.6)	8 (5.4)	17 (11.4)	24 (15.5)	102 (65.1)
13. On waking did you experience tremor?	6 (3.9)	20 (12.9)	20 (12.9)	44 (28.7)	65 (41.6)
14. Did you feel tired and sleepy after waking in the morning?	9 (5.8)	17 (11.3)	25 (16.4)	40 (25.8)	64 (40.6)
15. Did you wake up at night due to snoring or difficulties with breathing?	9 (5.8)	9 (5.8)	13 (8.7)	26 (16.8)	97 (62.9)

Univariate analysis was performed to determine factors associated with a PDSS-2 score > 18. A previous history of sleep disturbance before PD motor symptoms (OR 3.54; 95% CI 1.61–7.76, *p* = 0.001) and unemployment (OR 2.27; 95% CI 1.07–4.79, *p* = 0.023) both associated with a high PDSS-2 score. However a logistic regression analysis didn’t show a significant association with a previous history of sleep disturbance, age, gender, levodopa or trihexyphenidyl use, marital, educational or employment status.

**Fig. 1:**
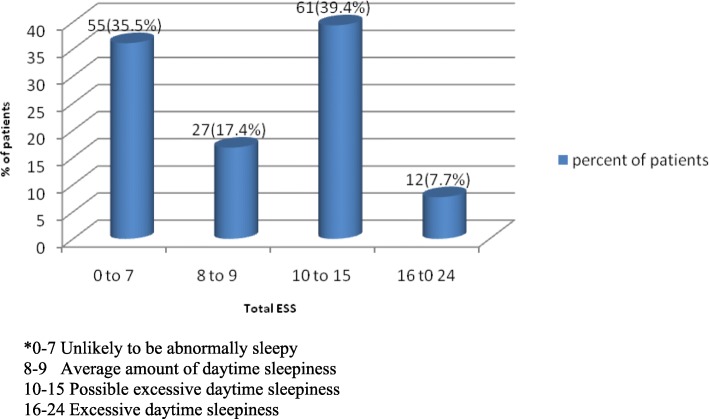
Frequency distribution of total Epworth sleepiness scale (ESS) scores*

The results of the ESS are shown in the Fig. 1. The median score was 9 (IQR 5–12). 73/155 (47.1%) of the patients had possible or definite excessive daytime somnolence. An EDSS score of > 10 associated with an H&Y score > 4 (*p* = 0.02). There was no statistically significant association between age, gender, PD duration, levodopa or trihexyphenidyl use, marital status, educational status or employment status with daytime sleepiness (EDSS score > 10).

On univariate analysis, there was a statistically significant association between ESS total score > 10 with only two variables of the PDSS-2: PD patients waking up at night due to snoring or difficulties with breathing (OR 2.87; 95% CI 1.25–6.60, *p* = 0.015) and getting up at night to pass urine (OR 2.3; 95% CI 1.08–4.92, *p* = 0.042). The other items of the PDSS-2 were not associated with a high ESS score.

## Discussion

We found that all of our Ethiopian PD patients reported some sleep problem, with a large minority (42.6%) reporting not sleeping well > 2 nights per week. One community based study from Norway reported that 60% of their PD patients had sleep problems [22].

Compared to patients from Germany [23] and the United Kingdom [24] in terms of overall sleep disturbance, there was a higher percentage of Ethiopian patients with a severe sleep disorder. Trenkwalder et al. [16] reported a mean PDSS score of 16.5- similarly to our mean score of 18.3 (median 17). However, they found that only 6.3% of their patients had a score > 30, whereas in our population, 23.2% of our patients had a score > 30 [16].

Nocturia and mobility difficulties were the most frequent sleep problems in our population. Other authors have found similar issues. Nocturia was reported by 62% of patients in the NMS Quest Study [25]. Adler et al. reported that 80% of patients with PD have two or more episodes of nocturia per night, and 33% urinate at least three times per night [26]. Lees and his colleagues [24] have reported nocturnal disturbances in 215 of 220 PD patients, including nocturia (79%) and difficulty turning over in bed (65%).

Over a third of our patients (36.1%) reported having distressing dreams. Nightmares have been reported in 30% of patients with PD and are correlated with disease severity and levodopa dose [27].

Insomnia occurs in about 30% of patients with PD. [27] Patients often develop a sleep pattern marked by excessive napping during the day and wakefulness at night [2]. We found sleep onset insomnia ≥2 days/week in 47 PD patients (30.3%) and sleep maintenance insomnia ≥2 days/week in 54 PD patients (34.9%). This is comparable with one study from India. Kumar et al. reported the prevalence of insomnia in PD patients were 30% [27].

Excessive day-time sleepiness (EDS) is a common complaint of patients with PD. [28, 29] It can occur early in PD [30], and may predate the diagnosis [31]. We found that 47.1% of our patients had possible or definite EDS. This is one of the highest rates reported in the world. Possible or definite EDS (ESS > 10) was seen in 15.5% of PD patients in Norway, 33% in Austria, 40.6% in New York USA, 46.2% in France, and 50.2% in Houston,USA [3, 32–35]. Adler et al. identified that advanced disease stage and age predicted EDS [26]. We also found an association between higher PD stage and higher ESS scores.

The number of patients using trihexyphenidyl is high (23.9%) in our study. This drug is not commonly used in western countries. However, due to cost, levodopa/carbidopa and trihexyphenidyl are the only available medications to treat PD symptoms. Therefore, trihexyphenidyl is often the first medication prescribed. Although its anticholinergic property may affect sleep, we did not find an association between trihexyphenidyl use and a higher level of sleep disturbance.

We found that high ESS scores associated with patients reporting both nocturia and breathing difficulties/snoring on the PDSS. OSA is defined as intermittently absent or reduced airflow during sleep despite respiratory effort. A study from Mexico City on 120 PD patients reported obstructive sleep apnea (OSA) in 39% of patients [36]. We found 57 PD patients (36.8%) reporting OSA symptoms at least 1 day per week. A study from France on 100 patients also reported 27% of PD patents were having obstructive sleep apnea [37].

One limitation of our study is that we did not assess body mass index (BMI) in our patients. High BMI is a major contributor of OSA. However, the prevalence of obesity in Ethiopia is very low. A World Bank report in June 2017 estimated the prevalence of obesity in Ethiopia to be less than 5% [38]. This was much lower than the other countries [38]. A summary report on risk factors for non-communicable diseases in Ethiopia from 2016 reported the prevalence of obesity (BMI > 30) for male and female Ethiopians was 0.5 and 2% respectively [39]. Another study from 2011 estimated the prevalence of obesity to be 2.1% for males and 10.2% for females. Although this study reported a higher rate among females than the others, this rate is still low, and most of our PD patients were male [40].

Another limitation of our study was that we did not assess for anxiety/depression. One study found an association between depression and sleep disorders [3], while others did not assess for depression or found no association [16, 23, 24, 31–34]. There is no validated depression scale for PD patients in Ethiopia, so we felt this was out of scope for this study.

Our study had other limitations. In Ethiopia there is no polysomnography (PSG), the gold standard for evaluating sleep disorders. Therefore, we had to rely on the PDSS-2. The PDSS is a subjective semi quantitative scale, which attempts to provide a holistic and clinical assessment of the complex etiology of sleep problems in Parkinson’s disease.

One other significant limitation of our study was our inability to assess for REM Sleep Behavior Disorder (RBD). Only 15 (9.7%) of our patients attended their clinic visits with a reliable sleep partner, so we could not use a questionnaire to evaluate for RBD, and of course, did not have access to PSG. In one study of 19 patients with PD, 47% met the diagnostic criteria of RBD based on PSG recordings, but only 33% of these cases were detected by a questionnaire [26]. We suspect that our percent of patients with Sleep Disorders would have been higher had we had a reliable way to assess for RBD.

## Conclusions

We found a higher percentage of Ethiopian patients with a high PDSS-2 score (> 18) than reported in other populations. Our patients also had one of the highest rates of EDS in the world. We cannot conclude from our data that this is due to poverty, but further investigation into this question is warranted.

## Data Availability

The data is on a password protected computer of Dr. DM. The datasets used and analyzed during the current study are available from the corresponding author upon reasonable request.

## Abbreviations

CI: Confidence Interval
EDS: Excessive Daytime Sleepiness
ESS: Epworth Sleepiness Scale
IQR: Inter Quartile Range
NMS: Non Motor Symptoms
PD: Parkinson ‘s Disease
PDSS-2: Parkinson’s Disease Sleep Scale Version 2
